# Calculation of specificity constants Γ for RUBISCO of different species from microcalorimetric data

**DOI:** 10.1007/s11120-025-01187-w

**Published:** 2025-12-01

**Authors:** Joachim Frank, Frank Müh

**Affiliations:** 1 Morsestrasse 11, 10587 Berlin, Germany; 2 Institut für Theoretische Physik, Johannes Kepler Universität Linz, Altenberger Strasse 69, Linz, A-4040 Austria

**Keywords:** Carbon dioxide, Enzyme activity, Isothermal titration calorimetry, Oxygen, Reaction enthalpy, Ribulose 1,5-bisphosphate carboxylase/oxygenase

## Abstract

Based on earlier data concerning the reaction enthalpy $\:{\varDelta\:}_{\mathrm{r}}H$ of Ribulose 1,5-bisphosphate carboxylase/oxygenase (RUBISCO) from spinach (Frank et al. Phys Chem Chem Phys 2:1301–1304, 2000), it is shown that the specificity constant $\:{\Gamma\:}$, indicating the ability of RUBISCO to discriminate between CO_2_ and O_2_ as substrate, can be determined from an isothermal titration calorimetric (ITC) measurement of $\:{\varDelta\:}_{\mathrm{r}}H$ as a function of the substrate concentration ratio $\:\rho\:=\left[\mathrm{C}{\mathrm{O}}_{2}\right]/\left[{\mathrm{O}}_{2}\right]$. The approach does not need any radioactive materials and might be a cost-effective alternative for RUBISCO engineering to screen for variants with a high specificity constant.

## Introduction

Ribulose 1,5-bisphosphate carboxylase/oxygenase (RUBISCO) is the key enzyme of the carbon reductive pathway, known as the Calvin-Benson-Bassham (CBB) cycle (Sharkey 2019; Zhao et al. 2024), and for photorespiration. It is a bifunctional enzyme that catalyzes both the carboxylation and oxygenation of the substrate D-ribulose 1,5-bisphospate (RuBP) by carbon dioxide (CO_2_) or oxygen (O_2_) at the same catalytic site (Cleland et al. 1998; Ogren 2003; Portis and Parry 2007; Prywes et al. 2023). RUBISCO of higher plants has a molecular weight of 550,000 Da and consists of eight large catalytic and eight small regulatory subunits (Andersson and Backlund 2008). The core of the hexadecameric enzyme of higher plants comprises four pairs of L-2 dimers which are similar in structure to the dimeric RUBISCO from *Rhodospirillum rubrum* (Knight et al. 1990). RUBISCO from higher plants shows more complex regulation properties (Lorimer 1981; Portis 1992), a higher carboxylase/oxygenase ratio (Jordan and Ogren 1981), and an apparent negative co-operativity behavior compared with the bacterial enzyme (Johal et al. 1985; Jensen and Zhu 1992). RUBISCO is activated in the presence of magnesium ions (Mg^2+^) and CO_2_ (Lorimer et al. 1976). In the presence of Mg^2+^ and CO_2_, all RUBISCO enzymes catalyze both the carboxylation and oxygenation of RuBP (Christeller 1981). Partitioning between both reactions is strongly influenced by divalent ions like Mg^2+^, Fe^2+^, Ni^2+^, Mn^2+^ and Co^2+^ (Christeller 1981). Transition metal ions strongly support the oxygenase reaction, while Mg^2+^ favours the carboxylation reaction (Christeller 1981). An important quantity in this respect is the specificity constant (or specificity factor) $\:{\Gamma\:}$ (defined below), which is a measure of the inherent ability of RUBISCO to discriminate between CO_2_ and O_2_ (Laing et al. 1974; Jordan and Ogren 1981). In the literature, this constant is also denoted as $\:{\Omega\:}$ (Tabita 1999), $\:\tau\:$ (Parry et al. 2003), $\:{S}_{r}$ (Uemura et al. 1997), or $\:{S}_{c/o}$ (Prywes et al. 2023; Aguiló-Nicolau et al. 2024). As is evident from the recent literature (Davidi et al. 2020; Bouvier et al. 2021; Aguiló-Nicolau et al. 2023), the determination of $\:{\Gamma\:}$ still relies largely on radioisotope labeling involving ^14^C and/or ^3^H (Zhu et al. 1992; Harpel et al. 1993; Kane et al. 1994). It has been noticed that these methods are inefficient and costly and pose limitations for high-throughput evaluation of RUBISCO variants in a biotechnological or agricultural context (Zhao et al. 2024). There are other methods for the determination of $\:{\Gamma\:}$ that do not require radioisotope labeling. These methods involve the measurement of O_2_ decline during the full consumption of RuBP using a Clarke-type electrode (Parry et al. 1989), quantification of the reaction products by anion-exchange chromatography (Uemura et al. 1996), nuclear magnetic resonance (NMR) spectroscopy (Wang et al. 1998), gas chromatography mass spectrometry (Whitney and Andrews 1998), and membane inlet mass spectrometry (Cousins et al. 2010). However, these methods appear to have not been widely implemented, which might be due to their complexity or systematic errors (Iñiguez et al. 2021).

In a former publication, we reported microcalorimetric measurements of the molar reaction enthalpy $\:{\varDelta\:}_{\mathrm{r}}H$ of the total RUBICSO reactions (Frank et al. 2000). Based on previously determined values of $\:{\Gamma\:}$ (Jordan and Ogren 1981, 1983), it was possible to derive separate values for the reaction enthalpies $\:{\varDelta\:}_{\mathrm{r},c}H$ of the carboxylase and $\:{\varDelta\:}_{\mathrm{r},o}H$ of the oxygenase reaction (Frank et al. 2000). Here, it is shown that the specificity constant Γ can be calculated from $\:{\varDelta\:}_{\mathrm{r}}H$, $\:{\varDelta\:}_{\mathrm{r},c}H$, and $\:{\varDelta\:}_{\mathrm{r},o}H$ independently. For this purpose, the reaction enthalpy $\:{\varDelta\:}_{\mathrm{r}}H$ of the total reaction has to be measured as a function of the concentration ratio of the gaseous substrates CO_2_ and O_2_. Therefore, we propose isothermal titration calorimetric (ITC) measurements as a possibly efficient screening method for RUBISCO variants with high specificity constants.

## Materials and methods

For completeness, we summarize here key aspects of the experiments performed earlier, whose details are described elsewere (Frank et al. 2000). The CO_2_ concentration was adjusted via the NaHCO_3_ concentration and pH of the solution. At pH 8.0, 40 mM NaHCO_3_ are in equilibrium with 0.6 mM CO_2_ (Hall et al. 1983). Stepwise dilution of NaHCO_3_ resulted in high and low CO_2_ concentrations. At low CO_2_ concentration below 10 µM, endogenous CO_2_ has to be taken into account. Boiling of solutions removes CO_2_. Resulting NaHCO_3_ (CO_2_) concentrations should be measured by a phosphoenolpyruvate carboxylase (PEPC) based assy (Hall et al. 1983). Another possibility is the use of gas syringes for mixing CO_2_ and O_2_ gas in appropriate ratios, followed by equilibrating the solutions. The main part of CO_2_ is dissolved as HCO_3_^−^, only traces exists as CO_2_ in solution (Hall et al. 1983). To circumvent low grades of activation, high concentrations of enzyme can be used (Frank et al. 2000). The overall reaction enthalpy $\:{\varDelta\:}_{\mathrm{r}}H\:$comprises both the carboxylase and oxygenase reaction catalyzed by RUBISCO. The molar enthalpy change $\:{\varDelta\:}_{\mathrm{r}}H$ is the sum of the evolved heat per mole of RuBP produced in the carboxylase and oxygenase reaction which contribute in a different manner (Frank et al. 2000). In the presence of Mg^2+^ as cofactor, the carboxylase reaction is favored in comparison to the oxygenase reaction at pH 8.0, 10 mM MgCl_2_ and 40 mM NaHCO_3_ (Christeller 1981). The ratio of the rates of carboxylase and oxygenase reaction is given by (Laing et al. 1974; Jordan and Ogren 1981)

1 $$\:\frac{{v}_{c}}{{v}_{o}}={\Gamma\:}\frac{\left[{\mathrm{C}\mathrm{O}}_{2}\right]}{\left[{\mathrm{O}}_{2}\right]}$$

with the specificity constant

2 $$\:{\Gamma\:}=\frac{{V}_{c}{K}_{o}}{{V}_{o}{K}_{c}}$$

Here, $\:{V}_{c}$ and $\:{V}_{o}$ are the maximal reaction rates for the carboxylase and oxygenase reaction, respectively, while $\:{K}_{c}$ and $\:{K}_{o}$ are the Michaelis-Menten constants for $\:C{O_2}$ and $\:{{O}}_{2}$, respectively. Let us denote the change of the RuBP (substrate) concentration due to turnover of the carboxylase activity by $\:\varDelta\:{\left[\mathrm{R}\mathrm{u}\mathrm{B}\mathrm{P}\right]}_{c}$ and correspondingly the change due the oxygenase activity by $\:\varDelta\:{\left[\mathrm{R}\mathrm{u}\mathrm{B}\mathrm{P}\right]}_{o}$. Then, it holds at a constant ratio

3 $$\:\rho\:=\frac{\left[{\mathrm{C}\mathrm{O}}_{2}\right]}{\left[{\mathrm{O}}_{2}\right]}$$

that (Frank et al. 2000)

4 $$\:\frac{\varDelta\:{\left[\mathrm{R}\mathrm{u}\mathrm{B}\mathrm{P}\right]}_{c}}{\varDelta\:{\left[\mathrm{R}\mathrm{u}\mathrm{B}\mathrm{P}\right]}_{o}}=\frac{{v}_{c}}{{v}_{o}}$$

If we define the part of RuBP converted in the carboxylase reaction as

5 $$\:x=\frac{\varDelta\:{\left[\mathrm{R}\mathrm{u}\mathrm{B}\mathrm{P}\right]}_{c}}{\varDelta\:{\left[\mathrm{R}\mathrm{u}\mathrm{B}\mathrm{P}\right]}_{c}+\varDelta\:{\left[\mathrm{R}\mathrm{u}\mathrm{B}\mathrm{P}\right]}_{o}}$$

we obtain

6 $$\:\frac{x}{1-x}=\frac{\varDelta\:{\left[\mathrm{R}\mathrm{u}\mathrm{B}\mathrm{P}\right]}_{c}}{\varDelta\:{\left[\mathrm{R}\mathrm{u}\mathrm{B}\mathrm{P}\right]}_{o}}=\frac{{v}_{c}}{{v}_{o}}={\Gamma\:}\rho$$

or, after rearrangement

7 $$\:x=\frac{{\Gamma\:}\rho\:}{1+{\Gamma\:}\rho\:}$$

The total reaction enthalpy $\:{\varDelta\:}_{\mathrm{r}}H$ of RUBISCO can be written as the weighted sum of the reaction enthalpies $\:{\varDelta\:}_{\mathrm{r},c}H$ and $\:{\varDelta\:}_{\mathrm{r},o}H$ of the carboxylase and oxygenase reaction, respectively, according to

8 $$\:{\varDelta\:}_{\mathrm{r}}H\:=x{\varDelta\:}_{\mathrm{r},c}H+\left(1-x\right){\varDelta\:}_{\mathrm{r},o}H$$

## Results

Taking together Eqs. (7) and (8), the molar reaction enthalpy $\:{\varDelta\:}_{\mathrm{r}}H$ of the total reaction can be calculated according to

9 $$\:{\varDelta\:}_{\mathrm{r}}H\:=\frac{{\Gamma\:}\rho\:}{1+{\Gamma\:}\rho\:}{\varDelta\:}_{\mathrm{r},c}H+\left(1-\frac{{\Gamma\:}\rho\:}{1+{\Gamma\:}\rho\:}\right){\varDelta\:}_{\mathrm{r},o}H$$

To calculate $\:{\varDelta\:}_{\mathrm{r}}H$, the values for $\:{\Gamma\:}$, $\:{\varDelta\:}_{\mathrm{r},c}H$, and $\:{\varDelta\:}_{\mathrm{r},o}H$ should be known. To demonstrate our procedure without additional measurements, we produced a dataset by calculating $\:{\varDelta\:}_{\mathrm{r}}H$ based on Eq. (9) for several values of $\:\rho\:=\left[\mathrm{C}{\mathrm{O}}_{2}\right]/\left[{\mathrm{O}}_{2}\right]$ (Table 1). $\:{\varDelta\:}_{\mathrm{r},o}H$ = – 401 ± 8 kJ/mol and $\:{\varDelta\:}_{\mathrm{r},c}H$ = – 62.3 ± 0.6 kJ/mol as determined earlier (Frank et al. 2000) are applied for RUBISCO from spinach along with known values of $\:{\Gamma\:}$ representing the status of RUBISCO with either Mg^2+^ or Mn^2+^ as cofactors. For RUBISCO from *R. rubrum*, the same molar reaction enthalpies $\:{\varDelta\:}_{\mathrm{r},c}H$ and $\:{\varDelta\:}_{\mathrm{r},o}H$ are chosen for illustrative purposes along with the appropriate values of $\:{\Gamma\:}$ for this species (Jordan and Ogren 1981, 1983).

**Table 1:** $\:{\varDelta\:}_{\mathrm{r}}H$ is calculated for several values of$\:\rho\:$with$\:{\varDelta\:}_{\mathrm{r},0}H$= – 401 ± 8 kJ/mole and$\:{\varDelta\:}_{\mathrm{r},c}H$= – 62.3 ± 0.6 kJ/mole (Frank et al. 2000) and different values of$\:{\Gamma\:}$obtained from the literature for RUBISCO from spinach and *R. rubrum* (Jordan and ogren 1981, 1983)

$\:-{\varDelta\:}_{\mathrm{r}}H$ (kJ/mol) $\:{\Gamma\:}=80$ Spinach, Mg^2+^	$\:-{\varDelta\:}_{\mathrm{r}}H$ (kJ/mol) $\:{\Gamma\:}=15$ *R. rubrum*, Mg^2+^	$\:-{\varDelta\:}_{\mathrm{r}}H$ (kJ/mol) $\:{\Gamma\:}=3$ Spinach, Mn^2+^	$\:-{\varDelta\:}_{\mathrm{r}}H$ (kJ/mol) $\:{\Gamma\:}=1.5$ *R. rubrum*, Mn^2+^	[CO_2_] (µM)	$\:\rho\:$
379	396.8	400.15	400.72	0.25	0.0008333
361	392.8	399.3	400.33	0.5	0.0016666
330	384.9	397.6	399.3	1	0.0033333
265.4	363.3	392.7	398.2	2.5	0.0083333
208	333.26	384.9	392.7	5	0.0166666
175	308.6	377.3	388.75	7.5	0.025
153	286.1	370.1	384	10	0.033333
106.2	212.8	333.2	363.3	25	0.083333
85	159.1	288	333.2	50	0.16666
78.1	133.6	255.7	308.4	75	0.25
74.5	118.7	231.5	288.1	100	0.33333
67.3	81.6	158.9	212.8	250	0.8333
64.8	75.3	118.6	158.8	500	1.6666
63.56	68.9	92.5	118.6	1000	3.3333

**Fig. 1:**
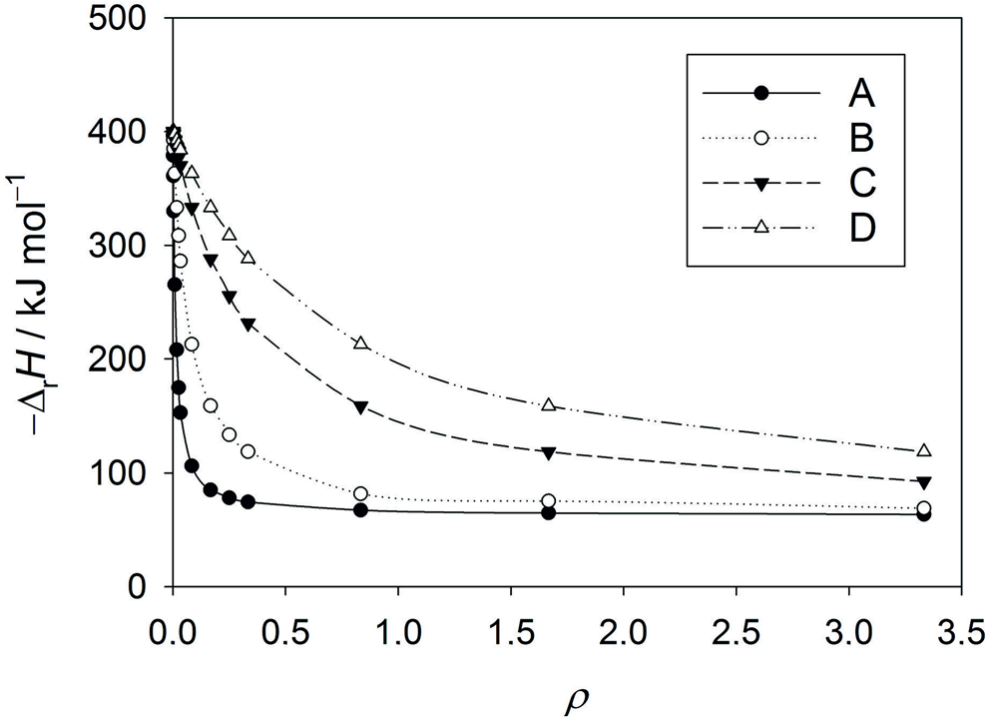
$\:{\varDelta\:}_{\mathrm{r}}H$ from Table 1 is displayed for several values of $\:\rho\:$. **A**: $\:{\Gamma\:}=80$ for spinach RUBISCO with Mg^2+^ as cofactor. **B**: $\:{\Gamma\:}=15$ for *R*. *rubrum* RUBISCO with Mg^2+^ as cofactor. **C**: $\:{\Gamma\:}=3$ for spinach RUBISCO with Mn^2+^ as cofactor. **D**: $\:{\Gamma\:}=1.5$ for *R*. *rubrum* RUBISCO with Mn^2+^ as cofactor. Values of $\:{\Gamma\:}$ obtained from the literature (Jordan and Ogren 1981; Jordan and Ogren 1983). Figure made with SigmaPlot 13 (© 2014 Systat Software Inc.)

Of course, $\:{\varDelta\:}_{\mathrm{r}}H$ can be measured by ITC for different fixed ratios $\:\rho\:=\left[\mathrm{C}{\mathrm{O}}_{2}\right]/\left[{\mathrm{O}}_{2}\right]$. A graph of $\:{\varDelta\:}_{\mathrm{r}}H$ as a function of $\:\rho\:$ allows to determine both molar reaction enthalpies $\:{\varDelta\:}_{\mathrm{r},c}H$, and $\:{\varDelta\:}_{\mathrm{r},o}H$. If one extrapolates [CO_2_] to infinity ($\:\rho\:\to\:\infty\:$), $\:{\varDelta\:}_{\mathrm{r}}H$ approaches $\:{\varDelta\:}_{\mathrm{r},c}H$. For [CO_2_] → 0 ($\:\rho\:\to\:0$), $\:{\varDelta\:}_{\mathrm{r}}H$ → $\:{\varDelta\:}_{\mathrm{r},o}H$. Both reaction enthalpies can be derived from a graph of $\:{\varDelta\:}_{\mathrm{r}}H$ as a function of $\:\rho\:$ (Fig. 1). Least square nonlinear regression fits yield also $\:{\varDelta\:}_{\mathrm{r},o}H$ und $\:{\varDelta\:}_{\mathrm{r},c}H$ at $\:\rho\:\to\:0$ and $\:\rho\:\to\:\infty\:$, respectively.

From Eq. (8) we learn that the part of RuBP converted in the carboxylase reaction $\:x$ can be expressed in terms of the reaction enthalpies according to

10 $$\:x=\frac{{\varDelta\:}_{\mathrm{r}}H-{\varDelta\:}_{\mathrm{r},o}H}{{\varDelta\:}_{\mathrm{r},c}H-{\varDelta\:}_{\mathrm{r},o}H}$$

Then, we can compute

11 $$\begin{aligned}&\:\frac{x}{1-x}=\frac{{\varDelta\:}_{\mathrm{r}}H-{\varDelta\:}_{\mathrm{r},o}H}{{\varDelta\:}_{\mathrm{r},c}H-{\varDelta\:}_{\mathrm{r},o}H}{\left(1-\frac{{\varDelta\:}_{\mathrm{r}}H-{\varDelta\:}_{\mathrm{r},o}H}{{\varDelta\:}_{\mathrm{r},c}H-{\varDelta\:}_{\mathrm{r},o}H}\right)}^{-1}\cr&\quad=\frac{{\varDelta\:}_{\mathrm{r}}H-{\varDelta\:}_{\mathrm{r},o}H}{{\varDelta\:}_{\mathrm{r},c}H-{\varDelta\:}_{\mathrm{r}}H}\end{aligned}$$

**Fig. 2:**
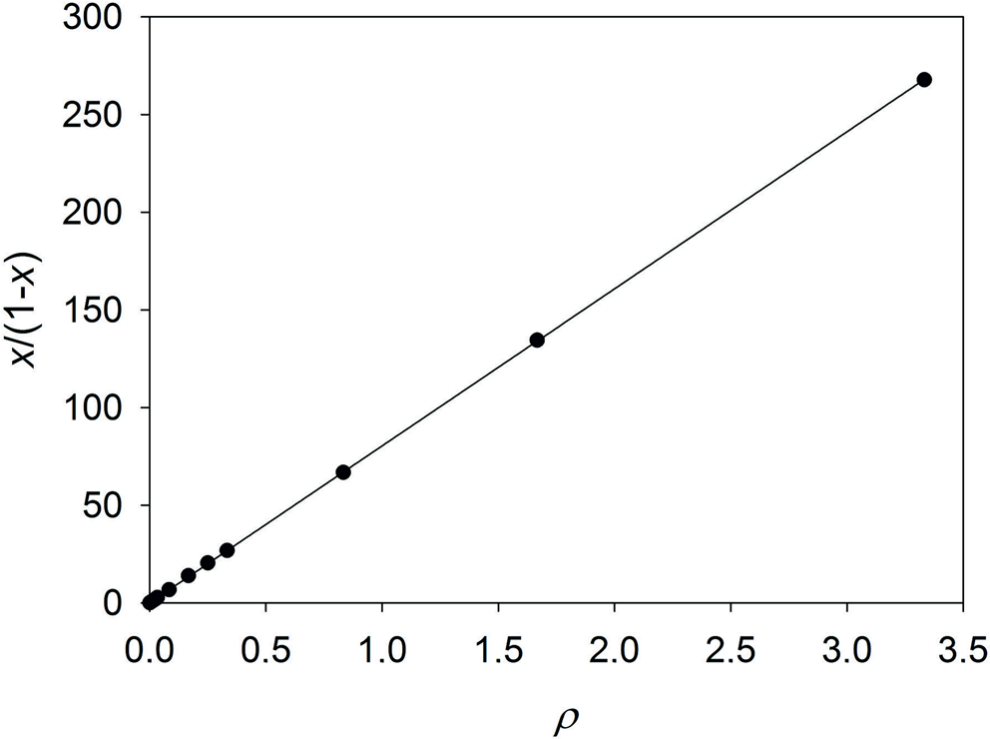
Linear graph of $\:x/(1-x)=$ $\left({\varDelta\:}_{\mathrm{r}}H-{\varDelta\:}_{\mathrm{r},o}H\right)/$ $\left({\varDelta\:}_{\mathrm{r},c}H-{\varDelta\:}_{\mathrm{r}}H\right)$ for RUBISCO of spinach as a function of $\:\rho\:=\left[\mathrm{C}{\mathrm{O}}_{2}\right]/\left[{\mathrm{O}}_{2}\right]$ with Mg^2+^ as cofactor. The specificity constant Γ can be calculated as the slope of the graph and is determined from linear regression (with zero intercept) to be 80.4 ± 0.1. Figure and fit made with SigmaPlot 13 (© 2014 Systat Software Inc.)

Thus, if $\:{\varDelta\:}_{\mathrm{r},o}H$ and $\:{\varDelta\:}_{\mathrm{r},c}H$ have been determined by extrapolation, it is possible to compute $\:x/(1-x)$ and plot it against $\:\rho\:$. According to Eq. (6), the slope of this plot is the specificity constant $\:{\Gamma\:}$. An example calculated from the values for $\:{\varDelta\:}_{\mathrm{r}}H$ of spinach in Table 1 and with $\:{\varDelta\:}_{\mathrm{r},o}H$ = – 401 kJ/mol and $\:{\varDelta\:}_{\mathrm{r},c}H$ = – 62.3 kJ/mol is shown in Fig. 2.

## Discussion

In a previous ITC study, the total molar enthalpy change $\:{\varDelta\:}_{\mathrm{r}}H$ associated with the carboxylase and oxygenase reaction catalyzed by RUBISCO of spinach was reported (Frank et al. 2000). Based on known ratios of the carboxylase and oxygenase reaction, the enthalpy changes $\:{\varDelta\:}_{\mathrm{r},c}H$ and $\:{\varDelta\:}_{\mathrm{r},o}H$ could be determined separately from $\:{\varDelta\:}_{\mathrm{r}}H$. For $\:{\varDelta\:}_{\mathrm{r},c}H$ of the carboxylase reaction predominatly catalyzed with Mg^2+^ as cofactor, a value of – 62.3 kJ/mol was obtained (Frank et al. 2000). For the oxygenase reaction, which is more efficiently catalyzed by Mn^2+^-activated RUBISCO from spinach than by the Mg^2+^-activated enzyme, a much higher negative reaction enthalpy of $\:{\varDelta\:}_{\mathrm{r},o}H$ = – 401 kJ/mol was determined (Frank et al. 2000). Obviously, the oxygenase reaction is an energy rich reaction, which probably proceeds via a peroxide intermediate (Bathellier et al. 2020). This is consistent with the finding that in the presence of Mn^2+^-ions, RUBISCO from spinach shows chemiluminescence during catalysis (Mogel and McFadden 1990), which is a good indicator for an energy rich process.

Here, we propose a two-step approach to determine the specificity constant Γ of RUBISCO by microcalorimetric measurements. The first step is to measure the molar reaction enthalpy $\:{\varDelta\:}_{\mathrm{r}}H$ for the carboxylase and oxygenase reaction and their different contribution to the total reaction as a function of the ratio $\:\rho\:=\left[\mathrm{C}{\mathrm{O}}_{2}\right]/\left[{\mathrm{O}}_{2}\right]$. This can be done by variation of the CO_2_ concentration at a fixed atmospheric O_2_ concentration of 0.3 mM at 25 °C. A graph of $\:{\varDelta\:}_{\mathrm{r}}H$ versus $\:\rho\:$ yields the value of $\:{\varDelta\:}_{\mathrm{r},c}H$ in the limit $\:\rho\:\to\:\infty\:$ and that of $\:{\varDelta\:}_{\mathrm{r},o}H$ in the limit $\:\rho\:\to\:0$. We demonstrated that the part of RuBP converted in the carboxylase reaction $\:x$ can be computed from the reaction enthalpy values. A plot of $\:x/(1-x)$ as a function of $\:\rho\:$ allows for a determination of the specificity constant Γ from the slope of the graph.

In a recent paper, Bloom and Kameritsch used ITC to investigate the thermodynamics of RUISCO metal binding (Bloom and Kameritsch 2017). However, to our knowledge, ITC has never been used to determine the specificity constant. In our earlier work (Frank et al. 2000), we also did not recognize this possibility. Nonetheless, it is apparent from Fig. 1 that due to the significant difference in reaction enthalpy between the carboxylase and the oxygenase reaction, which should be a conserved property of RUBISCO, the net reaction enthalpy becomes a function of the substrate ratio $\:\rho\:$. This dependence should enable a determination of Γ with errors that are determined by the accuracy of the calorimetric approach. In fact, microcalorimetric data are very precise. The experimental error is 2% for $\:{\varDelta\:}_{\mathrm{r},o}H$ and 1% for $\:{\varDelta\:}_{\mathrm{r},c}H$. Therefore, the calculation of $\:x/(1-x)$ from $\:{\varDelta\:}_{\mathrm{r}}H$, $\:{\varDelta\:}_{\mathrm{r},o}H$, and $\:{\varDelta\:}_{\mathrm{r},c}H$ for different $\:\left[\mathrm{C}{\mathrm{O}}_{2}\right]/\left[{\mathrm{O}}_{2}\right]$ ratios is very robust. In our earlier work (Frank et al. 2000), we used high protein concentrations to circumvent the effects of low activation grade. The ratio of RuBP/RUBISCO was between 1 and 2.5. Such high ratios are not necessary due the the catalytic properties of RUBISCO. We did not work out the minimum RUBISCO concentration, but high RUBISCO concentations lead to a quick response and a total conversion of RuBP.

In our view, the ITC approach has advantages over other approaches, since there is no need to use radioactive materials or to quantify the products of the carboxylation and oxygenation reaction. In particular, the latter procedure is often a source of errors in the determination of Γ (Iñiguez et al. 2021). We are presently not able to fully validate the ITC method against other approaches. Here, we merely show a proof of principle based on test data and hope that other researchers will take up the idea and perform further experiments for a benchmarking.

As a prospect, ITC could become an important tool for a screening of RUBISCO enzymes af various origin for high carboxylase/oxygenase reactions which may be useful to select and exploit plants with a highly efficient CO_2_-reduction pathway (Zhao et al. 2024). Analysis of their protein structure, in particular of their reaction centers, will be of high value to engineer plants with enhanced crop yields.

## Data Availability

All data are contained within the article.
